# Rectum necrosis in a patient with severe COVID19 infection after CAR-T therapy: a case report

**DOI:** 10.1186/s40792-024-02026-1

**Published:** 2024-09-26

**Authors:** Kiyoshi Saeki, Hidenobu Nakagama, Yuichi Tanaka, Yoshitaka Goto, Kazuhisa Kaneshiro, Hiroshi Kono, Kosuke Yanai, Hirofumi Yamamoto, Reiko Yoneda, Takashi Shimakawa, Takashi Ueki

**Affiliations:** 1 Department of Surgery, Hamanomachi General Hospital, 3-3-1, Nagahama, Fukuoka, 810-8539 Japan; 2 Department of Pathology, Hamanomachi General Hospital, Fukuoka, Japan; 3 Department of Hematology, Hamanomachi General Hospital, Fukuoka, Japan

**Keywords:** Rectum necrosis, COVID19, Coagulopathy, Emergency surgery

## Abstract

**Background:**

Coronavirus disease 2019 (COVID19) can cause gastrointestinal complications as well as respiratory tract disease. Coagulation abnormalities and thrombosis frequently occur in COVID19, especially in cases with severe clinical outcome. The relationship between gastrointestinal perforation and coagulopathy due to COVID19 remains unclear.

**Case presentation:**

A 49-year-old female received Chimeric antigen receptor T (CAR-T) therapy for an early recurrence of diffuse large B-cell lymphoma (DLBCL) that was refractory to chemotherapy. She was diagnosed with cytokine release syndrome (CRS) because of a fever and oxygen desaturation, and administered tocilizumab. Forty days after completing CAR-T therapy, she was infected with COVID19 and transferred to our hospital. Her general condition worsened and she developed COVID19 pneumonia, and then steroid pulse therapy was started. While her respiratory condition improved, she experienced pain in the anal region and computed tomography (CT) revealed a rectal perforation. An emergency surgery was undertaken, and the lower rectum wall was found to be completely necrotic. Removal of the necrotic part of the rectum tissue, and drainage and lavage of necrotic tissue in the pelvic cavity were performed. The remaining rectum was resected with partial sigmoidectomy, but we could not make the anal stump closed. In addition, an end colostomy in the sigmoid colon was performed. Histopathological findings showed thromboses in the rectal mesentery veins. After the first surgery, the pelvic abscess cavity persisted and her high-grade fever continued. Reoperation was laparoscopically performed, and she underwent a resection of anal canal with residual necrotic rectal and mesorectal tissue, and a drainage of the pelvic abscess. After the reoperation, her general condition improved and CT showed that the abscess cavity had significantly improved.

**Conclusions:**

Gastrointestinal perforation, especially rectal necrosis due to coagulopathy caused by severe COVID19 infection, is a rare but life-threatening complication. Physicians should have a high degree of clinical suspicion for timely diagnosis and management, and surgical intervention is necessary in cases of rectal necrosis.

**Supplementary Information:**

The online version contains supplementary material available at 10.1186/s40792-024-02026-1.

## Background

Coronavirus disease 2019 (COVID19) caused by the severe acute respiratory syndrome coronavirus 2 (SARS-CoV-2) is a newly emerging infectious disease that caused a global pandemic beginning in December 2019 [1]. Although most patients with COVID19 predominantly have a respiratory tract infection, gastrointestinal symptoms are also a common complication [2], and some cases of gastrointestinal perforation caused by COVID19 have been reported [3–12].

Chimeric antigen receptor T-cell (CAR-T) therapy is a promising form of immunotherapy that uses genetically modified T cells to target cancer cells and demonstrates impressive efficacy against hematologic malignancies [13]. Patients receiving immunotherapy, including CAR-T therapy, for cancer treatment exhibit more severe COVID19 clinical outcomes and often develop a cytokine storm [14]. Thrombotic events are common complications in COVID19 patients, and cytokine storm caused by severe COVID19 infection leads to abnormal coagulopathy and a prothrombotic state [15].

We herein report the case of a patient with rectum necrosis who had severe COVID19 infection after CAR-T therapy for a recurrence of diffuse large B-cell lymphoma (DLBCL), and who survived postoperatively. Histopathological findings indicated that the rectum necrosis was caused by thromboses in the rectal mesentery veins.

## Case report

A 49-year-old female was diagnosed with CD5-positive DLBCL Stage IV and underwent chemotherapy based on an R-CHOP-like regimen [16] for about 9 months. One month after completing the chemotherapy, she became aware of a right breast mass. The mass was diagnosed as DLBCL recurrence by needle biopsy, and positron emission tomography (PET–CT) showed intensive fluoro-deoxyglucose (FDG) accumulation of multiple lymph nodes throughout the body and multiple bone involvement. There was no improvement of the DLBCL recurrence by a salvage chemotherapy (ESHAP regimen). CAR-T therapy was performed because the mass was an early recurrence of DLBCL that was refractory to chemotherapy.

On the fourth day after the CAR-T therapy, a fever and low oxygen were found, and she was diagnosed with cytokine release syndrome (CRS) grade 2. After administering tocilizumab (8 mg/kg, twice), the CRS symptoms improved. One month later after completing the CAR-T therapy, PET–CT showed no intensive FDG accumulation of multiple lymph nodes throughout the body, and she achieved a complete response to it and the remission of DLBCL.

Forty days after completing CAR-T therapy, the patient was infected with COVID19. She was treated with remdesivir, and urgently hospitalized for vomiting, diarrhea and loss of consciousness. She spiked a high-grade fever (38 ℃), and was tachycardiac to 100 beats/min and hypotensive to 80/50 mmHg. Laboratory workup showed neutropenia with a white blood cell (WBC) count of 100/μL (10.6% neutrophil count) (Supplementary Table 1 and Supplementary Fig. 1), and she was treated with broad-spectrum antibiotics and granulocyte colony stimulating factor (G-CSF). Bone marrow analysis led to a diagnosis of macrophage activation syndrome (MAS), and she was started on steroids (weight-based methylprednisolone 2 mg/kg per day).

**Fig. 1:**
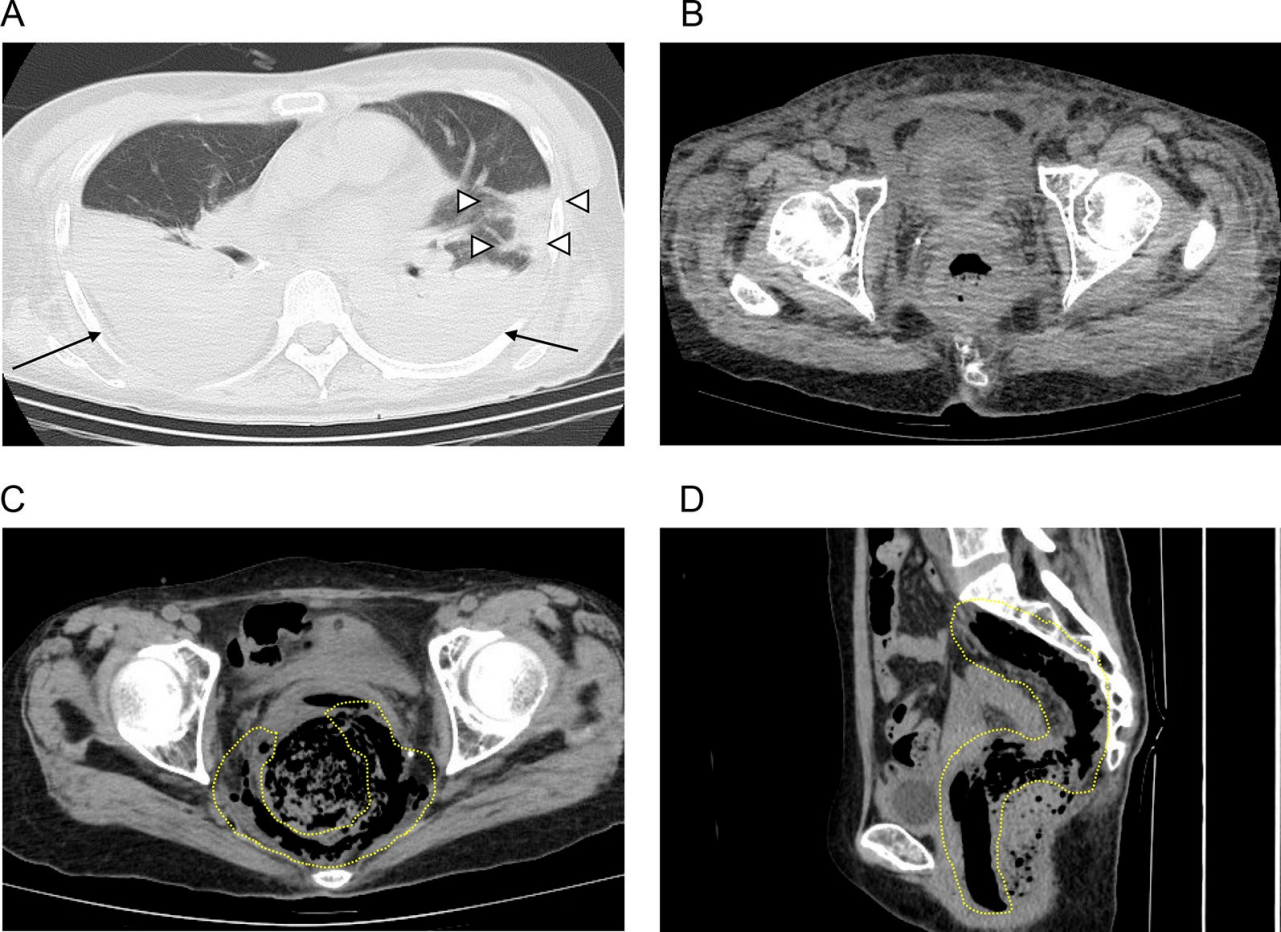
**A, B** CT imaging findings during COVID19 pneumonia. **A** Bilateral pleural effusion and a consolidation surrounded by ground-glass opacity in the left lung predominance were observed. Arrows indicate the pleural effusion. White arrowheads indicate the consolidation. **B** Rectum was slightly edematous. **C, D** CT imaging findings before emergency surgery. **C **(axial section) and **D** (sagittal section): free air was observed in the pelvic cavity. A yellow dotted line indicates the free air

An 8 L/min oxygen mask was required to maintain her oxygen saturation over 90%, and computed tomography (CT) showed bilateral pleural effusion and a consolidation surrounded by ground-glass opacity in the left lung predominance (Fig. 1A). She was given a diagnosis of COVID19 pneumonia. The rectum was slightly edematous but there was no evidence of thromboembolism by CT (Fig. 1B). After initiation of steroid pulse therapy (500 mg twice daily for 3 days) her respiratory condition improved (saturation greater than 96% in room air).

Three days after the start of steroid pulse therapy, she became aware of the slight pain in the anal region, which exceeded gradually day by day, and 7 days after her first awareness anal pain a CT scan revealed free air in the pelvic cavity (Fig. 1C, D). An emergency surgery was conducted under a preoperative diagnosis of rectum perforation.

**Fig. 2:**
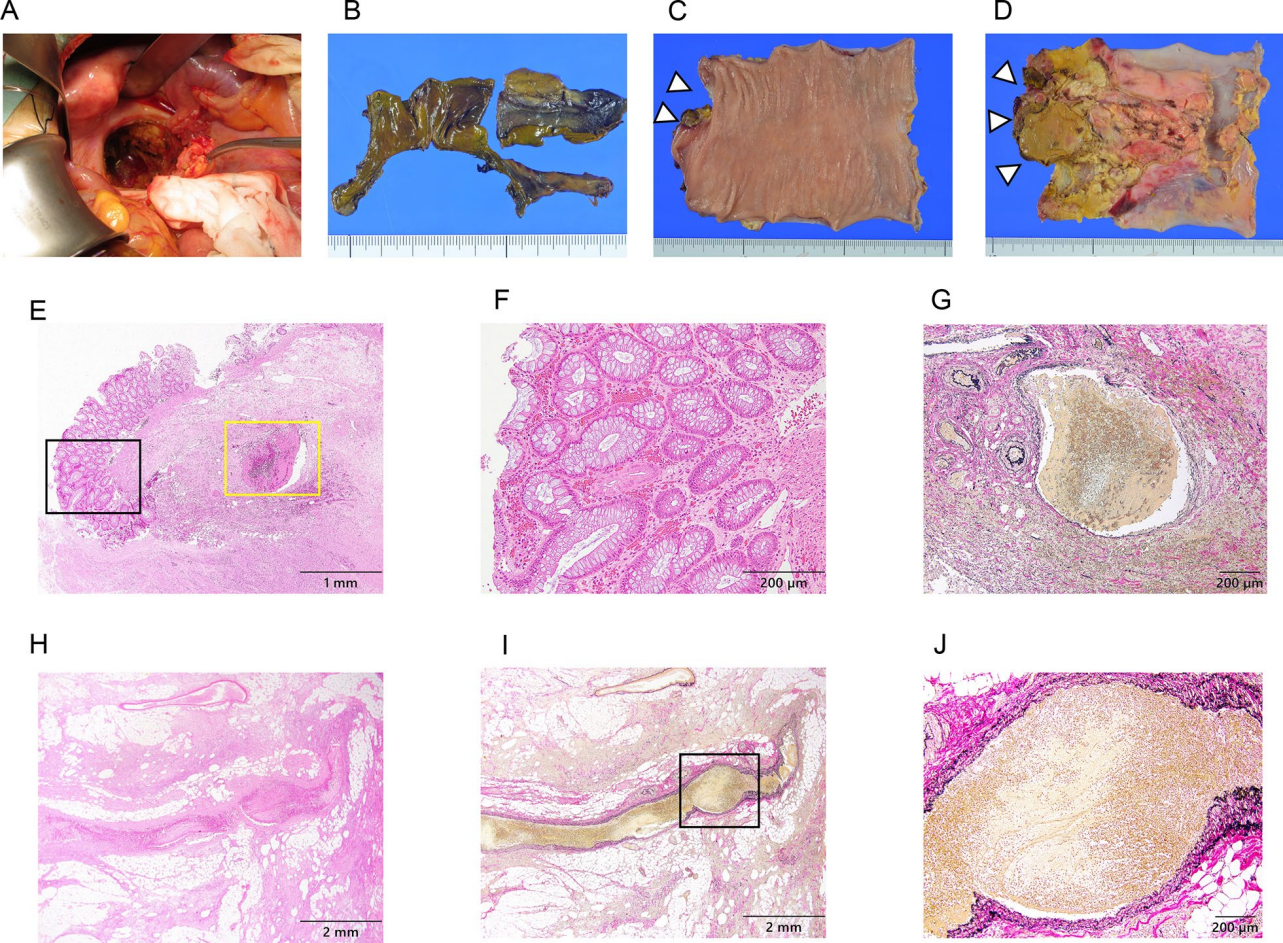
**A** Operative findings during emergency surgery. **A** Lower rectum necrotic tissue in the pelvic cavity was drained. **B–D** Macroscopic findings of the resected specimen. **B** Lower rectum tissues were completely necrotic. **C**, **D** On the resected upper rectum closed to the anus side, the mucous side surface had slight ischemic change (**C**), and the serosal side surface had ischemic change (**D**). White arrowheads indicate the macroscopic slightly ischemic mucous side surface lesion of the area shown in **E–G**, and the macroscopic ischemic serosal side surface lesion of the area in **H–J. E–J** Pathological findings of the resected rectum specimen. Venous thromboses were observed in the mesenteric vein. **E** Low magnification image of the resected upper rectum closed to the anus side of the mucous side surface by hematoxylin and eosin (H.E.) staining. **F** High magnification image of the black frame rectangle inset of **E**. Hemorrhage and frequent thrombi were observed in the mucosal layer by H.E. staining. **G** High magnification image of the yellow frame rectangle inset of **E**. An approximately 1 mm diameter mesenteric venous thrombosis was observed in the proper muscular layer by Elastica van Gieson (EVG) staining. **H, I** Low magnification image of the resected upper rectum closed to the anus side of the serosal side surface by H.E. staining (**H**) and by EVG staining (**I**). **J** High magnification image of the black frame rectangle inset of (**I**) by EVG staining. An over 1 mm diameter mesenteric venous thrombosis was observed in the subserosal layer by EVG staining

The operation was performed as follows: the lower rectum wall extending to the anal canal was completely necrotic (Fig. 2A), and a 1.5 cm perforated site was found on the back wall of the vagina.

Removal of the necrotic part of the rectum tissue and abdominal and pelvic drainage were carried out.

Although we excised as much necrotic part of the rectum tissue as possible, we could not set the resection line on the anal side of the rectum because the rectum was completely necrotic and the necrosis extended all the way to the anal canal. Therefore, we could not make the anal stump closed. Considering the patient’s poor general condition, we decided to perform minimum surgery in an emergency setting. The remaining rectum was resected with partial sigmoidectomy, and an end colostomy in the sigmoid colon was performed. One closed drain was placed in the abdomen and two closed drains were transabdominally placed in the pelvis. To separate the abdomen from the pelvis, the pelvic cavity was closed using uterus, bilateral oviduct, peritoneum and adipose tissue. Grossly, the lower rectum was completely necrotic (Fig. 2B), and in the resected upper rectum closed to the anus side, the mucous side surface had slight ischemic change (Fig. 2C), and the serosal side surface had ischemic change (Fig. 2D). In the histopathological findings, on the resected upper rectum closed to the anus side, hemorrhage and frequent thrombi were observed in the mucosal layer (Fig. 2E, F) and an approximately 1 mm diameter venous thrombosis was observed in the proper muscular layer (Fig. 2E, G). In addition, a mesenteric venous thrombosis of more than 1 mm diameter was recognized in the subserosal layer (Fig. 2H, I).

**Fig. 3:**
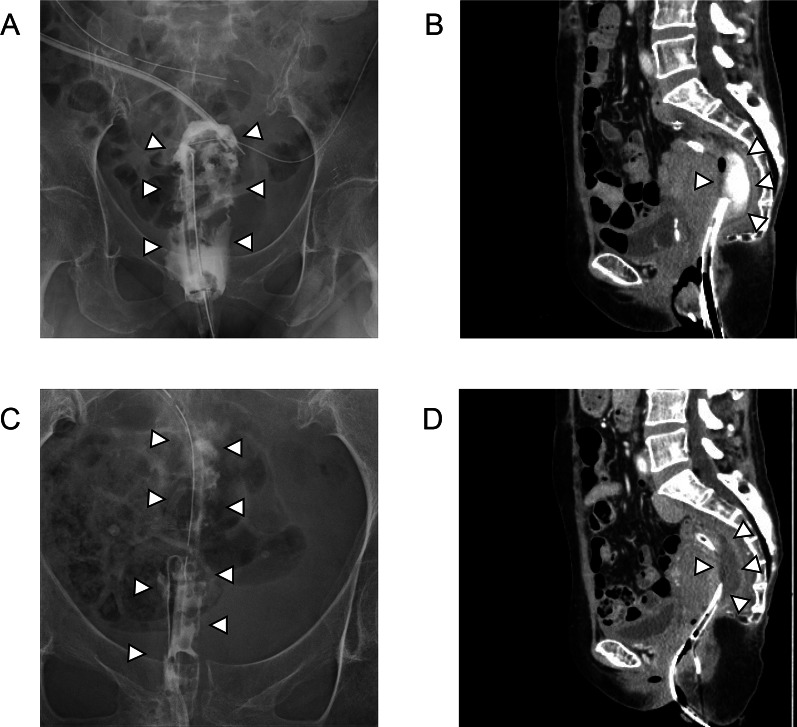
**A, B** Radiographic contrast examination (**A**) and CT imaging (**B**) findings after the first surgery. **A** Transanal drainage tube was added to the pelvic abscess cavity. The abscess cavity was contrasted by gastrografin. **B** CT image findings indicated a pelvic abscess cavity. **C**, **D** Radiographic contrast examination (**C**) and CT imaging (**D**) findings before reoperation. **C, D** Pelvic abscess cavity decreased but persistently remained by radiographic contrast examination (**C**) and CT (**D**). White arrowheads indicate the abscess cavity

For several days after the surgery, her general condition improved. However, purulent discharge was observed from the drain placed in the pelvis and she developed a high-grade fever. In addition to the antibiotics therapy, a transanal drainage tube to the abscess cavity was added (Fig. 3A, B). Using both the transanal drain and the pelvic drains, the continuous lavage with saline was performed. The size of the pelvic abscess cavity decreased, but the cavity persisted (Fig. 3C, D) along with the high-grade fever. Based on the clinical course, we explained the patient that reoperation should be performed. However, the patient and her family requested the second opinion to another hospital, and the medical doctor in the hospital insisted on continuing the antibiotics therapy and the continuous lavage with saline using the drains rather than surgery. Although we continued them, her purulent discharge persisted with high-grade fever. We explained the necessity of reoperation to her and her family again. Eventually, she decided to receive a reoperation.

**Fig. 4:**
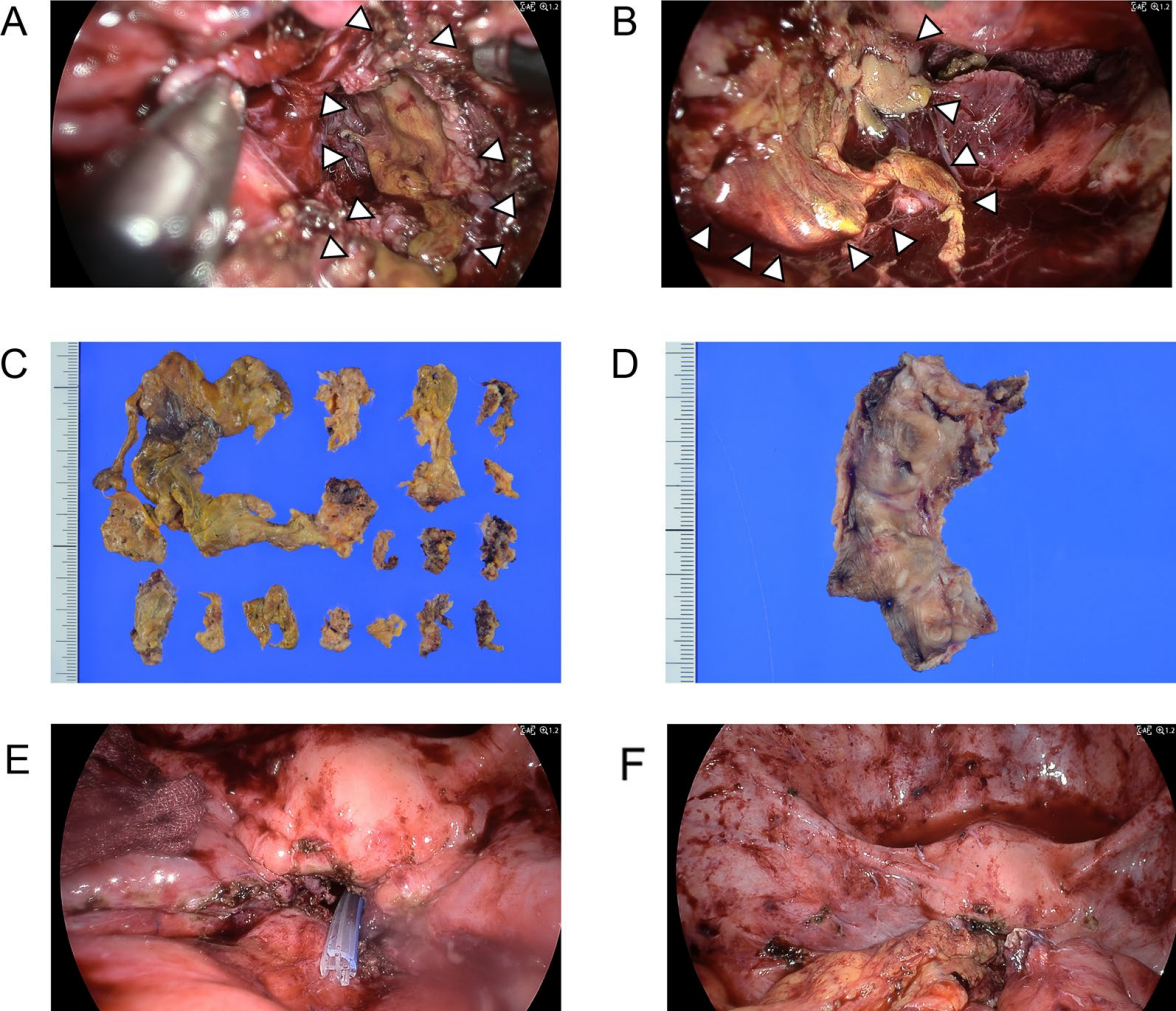
**A**, **B** Operative findings during second surgery. **A**, **B** Necrotic tissue, which was thought to be residual rectum and mesorectum, was observed in the pelvic abscess cavity. **C**, **D** Macroscopic image findings of the resected specimen. **C** Macroscopic findings of the resected residual rectal and mesorectal necrotic tissue. **D** Macroscopic findings of the resected anus and anal canal. **E**, **F** Operative findings during reoperation. **E** Closed drain was placed in the pelvic cavity. **F** Omental pedicle flap was packed in the pelvic cavity. White arrowheads indicate the residual rectal and mesorectal necrotic tissue

Reoperation was performed laparoscopically 146 days after the initial surgery as follows: necrotic tissue, which was thought to be residual rectum and mesorectum, was observed in the abscess cavity (Fig. 4A, B). She underwent a resection of anal canal with residual necrotic rectal and mesorectal tissue, and a drainage of the pelvic gangrenous tissue from both sides of the abdomen and perineum (Fig. 4A–D). A closed drain was placed in the pelvic cavity (Fig. 4E) and the cavity was packed with an omental pedicle flap (Fig. 4F).

Nine days after the second surgery, the closed drain placed in the pelvic cavity was removed. 2 days later, she developed a high-grade fever and a transvaginal drain was placed into the pelvic cavity with purulent discharge from it, and she was treated with broad-spectrum antibiotics. Her fever gradually went down to normal and the purulent discharge from the drain decreased. 20 days after the reoperation, the transvaginal drain was removed. After pain control medication, she was discharged home on the 43rd day after the second surgery, and a follow-up CT 100 days after the reoperation showed that the abscess cavity had significantly improved.

## Discussion

Gangrenous ischemic proctitis is extremely rare because of the abundance of collateral vessels in the rectum [17–20]. The blood supply of the rectum derives from the inferior mesenteric, internal iliac, and internal pudendal arteries. Ischemic gangrenous proctitis is mainly described in patients with significant atherosclerotic and cardiac risk factors in the setting of hemodynamic instability [18–20]. However, our patient did not have any such risk factors. Aside from arterial occlusion, venous insufficiency can also lead to ischemia and necrosis of the tissue, since the rectum has abundant venous drainage to the portal and caval systems [17]. In earlier reports of COVID19-related gastrointestinal perforations, both upper [3] and lower gastrointestinal perforations [3–12, 21–24] have been described. There are 4 cases of rectal perforation related to COVID19 [22–24]. Two of these 4 patients went into septic shock on the day of hospitalization, and CT showed rectal wall thickening with free air around the mesorectum and an inflammatory stranding of perivisceral fat tissue; both these patients died without surgery within 24 h after hospitalization [23, 24]. One of the remaining 2 patients had a 1 cm diameter perforation over the anterior wall of the upper one-third of the rectum, which was repaired and a decompressing transverse loop colostomy was performed; this patient survived postoperatively [22]. In the other patient, CT showed rectal wall thickening surrounded by extraluminal free air with a small-sized fluid collection; this patient was treated conservatively and survived [24]. While conservative management is appropriate in early cases or cases with small perforations with superficial ischemia, rectal perforation cases related to COVID19 have a high mortality rate and require emergency surgery.

In our present case, the lower rectum and the surrounding mesorectum were completely gangrenous and the upper rectum was partially necrotic. The patient underwent emergency surgery and survived postoperatively. Then she underwent a resection of anal canal with residual necrotic rectal and mesorectal tissue at the reoperation and was discharged from the hospital without severe complications.

Mesenteric venous thrombosis is often caused by acquired hypercoagulable states, inherited disorders, and intra-abdominal traumatic or inflammatory conditions [25]. Many studies have reported that SARS-CoV-2 can lead to a prothrombotic state, reflecting the high cumulative incidence of associated thrombotic events, particularly in patients receiving intensive care [26, 27]. Severe COVID19 is associated with increased levels of proinflammatory cytokines, and cytokine storm initiates a prothrombic condition [15, 27]. There are some reports of mesenteric venous thrombosis related to COVID19 infection [28, 29]. In the reports of gastrointestinal perforation occurring in COVID19 patients, vascular thrombosis in the mesenteric vein was histologically recognized, and it was proposed that mesenteric venous thrombosis caused the gastrointestinal perforation [6, 10–12, 21].

In our case, the patient was in a highly prothrombotic state due to a cytokine storm resulting from severe COVID19 infection, which may have caused the mesenteric venous thrombosis. Histologically, thromboses of over 1 mm diameter in the mesentery veins were recognized in the rectum close to the rectal necrotic tissue. Because the distal rectum is usually spared due to its collateral blood supply, it may not be clear that the cause of rectal necrosis was limited to mesenteric venous thrombosis. Not only mesenteric venous thromboses, but also hemodynamic instability derived from severe COVID19 infection may cause ischemia of the entire rectal collateral blood supply. Otherwise, initially, a relatively small rectal perforation caused by rectal mesenteric venous thrombosis may have gradually worsened to rectal necrosis due to the delay in the clinical expression of peritonitis. However, we believe that mesenteric venous thromboses were the main trigger of rectal ischemic necrosis in this case.

CAR-T therapy is a promising immunotherapeutic approach and is currently used for several hematologic malignancies [13]. CAR-T therapy carries the risk of inflammatory toxicities, such as CRS [30, 31]. In patients with progressive hypotension or hypoxia because of CRS, the IL-6 receptor antagonist tocilizumab has been the primary therapy [32, 33]. Tocilizumab has also been used for people with severe COVID19 infection [34, 35]. To date, there have been no reports of a direct relationship between CAR-T therapy and gastrointestinal perforation. However, gastrointestinal perforation has been described as a rare but potentially severe complication of tocilizumab, although the pathophysiology underlying this relation is poorly understood [36–39]. Several cases of intestinal perforations in COVID19 patients administered tocilizumab have been reported [3, 4, 39]. In regard to steroid therapy, it has been reported that corticosteroid use was associated with increased risk of gastrointestinal perforation [40–42]. Corticosteroids are well known to inhibit wound repair via global anti-inflammatory effects and suppression of cellular wound responses, and to increase the risk of wound infection [43]. High-dose corticosteroids such as steroid pulse therapy have been reported to decrease the clinical expression of peritonitis to the point that recognition and, therefore, treatment of gastrointestinal perforation were markedly delayed, resulting in high mortality rates [40]. Cases of intestinal perforation cases in COVID19 patients receiving steroid therapy have also been reported [3, 4, 8, 9]. Patients with baseline immunosuppression with cancer receiving immunotherapy regimen including CAR-T therapy had worse COVID19 severity and cytokine storm [14].

In our case, cytokine storm and prothrombic condition were initiated by severe COVID19 infection after CAR-T therapy. COVID19-associated coagulopathy triggered the thromboses in the mesenteric vein and eventually caused rectal ischemic necrosis. In addition, tocilizumab may have facilitated the rectal perforation. Although there was no direct relationship between the CAR-T therapy and the patient’ rectal necrosis, we speculated that the CAR-T therapy was affecting the patient’s clinical course to some extent. Moreover, the wound healing-suppressive and infectious effects of steroid therapy may have exacerbated the rectal perforation derived from rectal mesenteric venous thrombosis, while the steroid pulse therapy with high-dose corticosteroids for COVID19 pneumonia could have delayed a proper diagnosis by mitigating the clinical presentation of anus pain, eventually led to worsening of the rectal necrosis.

Rectal necrosis is a life-threatening disease and emergency surgery is the only option [18–20]. The surgical management of rectal necrosis is controversial and depends on the clinical status of the patient and the surgical findings. Maun et al. [18] proposed that complete proctectomy with end colostomy may be necessary as a life-saving measure in cases in which the ischemia and gangrene involve the entire rectum all the way to the dentate line, because the retention of dead rectum may create a persistent source of sepsis. Azimuddin et al. [20] reported 3 cases with ischemic gangrene of the rectum, 2 of which were surgically found to have complete gangrene of the rectum extending to the dentate line, and abdominoperineal resections (APRs) were performed. In the other patient, severe gangrene of almost the entire rectum except the last 2 cm above the dentate line was found, and because the anal canal was viable, the patient was able to avoid APR and instead underwent a low anterior resection with Hartman’s procedure. The surgeon considered that leaving behind a gangrenous rectal segment would create a source of persistent sepsis and thus the segment should be removed. On the other hand, in a report by Nassif et al. [19], a patient with necrosis of the rectum and anus with absent sphincter tone as well as extensive necrosis of the descending and sigmoid colon received a transverse colostomy and mucous fistula, leaving the gangrenous descending and sigmoid colon with the rectum in place. Nassif et al. contended that APR appears to be associated with more severe mortality in an emergency setting and would add substantial risk to the primary procedure.

In our case, if the anal canal had been viable at the emergency surgery, we could have stapled off and divided the anorectal stump, and performed a Hartman’s procedure. However, the rectum was completely necrotic, and the necrosis extended all the way to the anal canal, and we only performed the removal of rectal necrotic tissue and resection of the remaining rectum with end colostomy. After the emergency surgery, the patient’s recovery was prolonged and eventually she underwent a resection of anal canal with residual necrotic rectal and mesorectal tissue because the remaining gangrenous rectal segment had become a source of persistent purulence. It is not clear whether we should carry out APR even at the emergency surgery, but in our case the patient was in an unstable condition due to severe COVID 19 infection. Therefore, we avoided APR at emergency surgery, and the patient survived postoperatively and was finally discharged from the hospital without severe complications.

## Conclusions

We reported a case of rectal necrosis caused by thrombosis initiated by severe COVID19 after CAR-T therapy. Tocilizumab and high-dose corticosteroid are indicated in patients with severe COVID19 or CRS after CAR-T therapy, and both therapies increase the risk of gastrointestinal perforation. Rectal necrosis is a rare but life-threatening complication of COVID19 infection, especially for the patients with baseline immunosuppression after an immunotherapy regimen that includes CAR-T therapy. Surgical management is necessary in case of gangrenous proctitis, and we were able to save our patient’s life by performing the surgery in two stages to avoid excessive invasiveness.

## Supplementary Information

Supplementary Material 1.

Supplementary Material 2.

Supplementary Material 3.

## Data Availability

Data sharing is not applicable to this article as no datasets were generated or analysed during the current study.

## Abbreviations

COVID19: Coronavirus disease 2019
CAR-T: Chimeric antigen receptor T
DLBCL: Diffuse large B-cell lymphoma
CRS: Cytokine release syndrome
CR: Complete response
CT: Computed tomography
SARS-CoV-2: Severe acute respiratory syndrome coronavirus 2
PET–CT: Positron emission tomography
FDG: Fluoro-deoxyglucose
G-CSF: Granulocyte colony stimulating factor
MAS: Macrophage activation syndrome
H.E.: Hematoxylin and eosin
EVG: Elastica van Gieson
APR: Abdominoperineal resection
