# Supplementary material for: Rectum necrosis in a patient with severe COVID19 infection after CAR-T therapy: a case report
Source: Surg Case Rep. 2024 Sep 26;10:227. doi: 10.1186/s40792-024-02026-1 (PMC11427651; doi:10.1186/s40792-024-02026-1)
Supplement: Supplementary file 1 — Supplementary Material 1. [file 40792_2024_2026_MOESM1_ESM.pptx]

## Slide 1
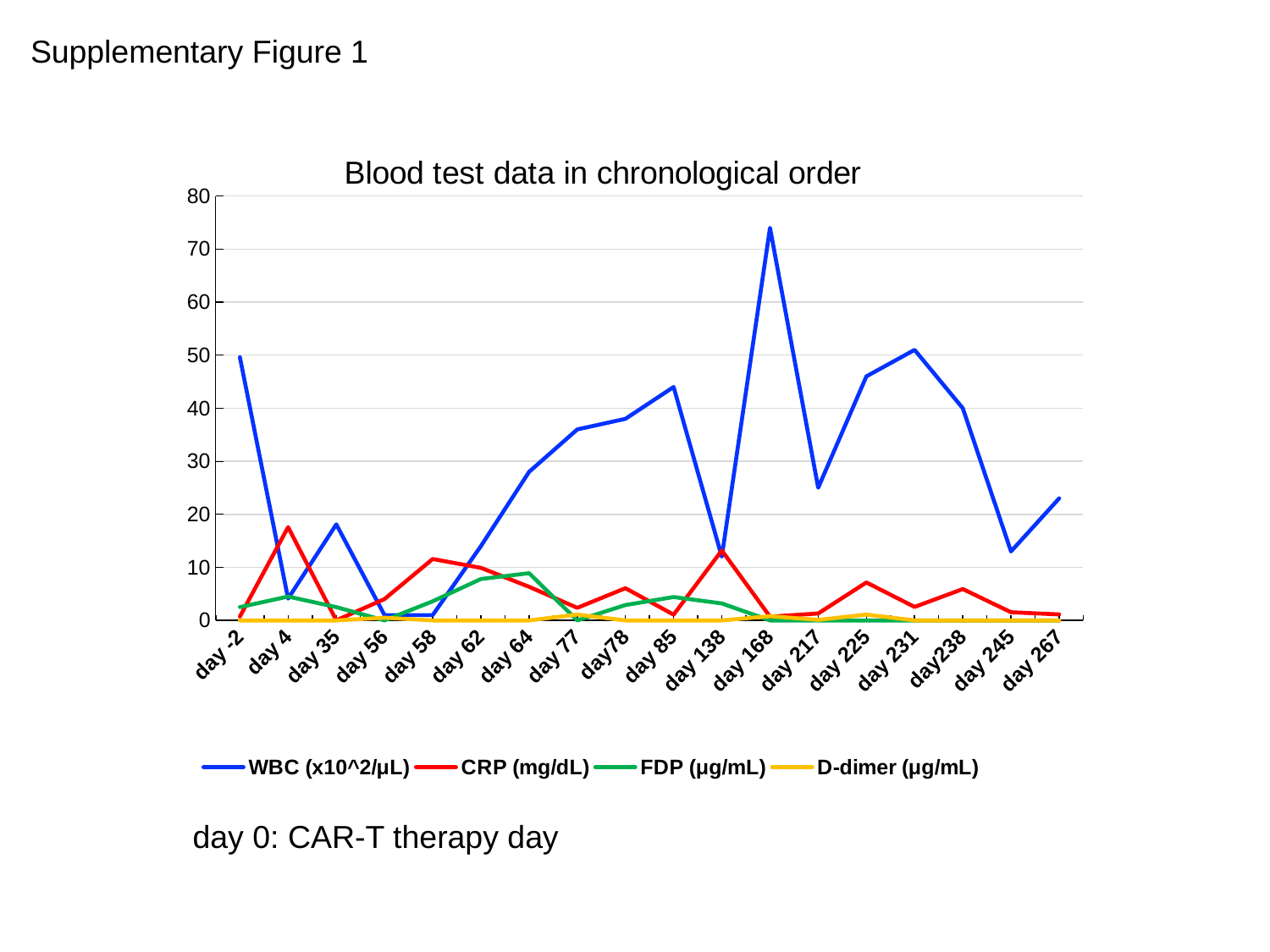

Supplementary Figure 1
### Chart: Blood test data in chronological order
| Category | WBC (x10^2/μL) | CRP (mg/dL) | FDP (μg/mL) | D-dimer (μg/mL) |
|---|---|---|---|---|
| day -2 | 49.6 | 0.73 | 2.5 | 0.0 |
| day 4 | 4.1 | 17.57 | 4.5 | 0.0 |
| day 35 | 18.1 | 0.01 | 2.5 | 0.0 |
| day 56 | 1.0 | 4.02 | 0.0 | 0.5 |
| day 58 | 1.0 | 11.57 | 3.6 | 0.0 |
| day 62 | 14.0 | 9.9 | 7.8 | 0.0 |
| day 64 | 28.0 | 6.33 | 8.9 | 0.0 |
| day 77 | 36.0 | 2.37 | 0.0 | 1.1 |
| day78 | 38.0 | 6.07 | 2.9 | 0.0 |
| day 85 | 44.0 | 1.08 | 4.4 | 0.0 |
| day 138 | 12.0 | 13.18 | 3.2 | 0.0 |
| day 168 | 74.0 | 0.71 | 0.0 | 0.8 |
| day 217 | 25.0 | 1.3 | 0.0 | 0.1 |
| day 225 | 46.0 | 7.15 | 0.0 | 1.1 |
| day 231 | 51.0 | 2.54 | 0.0 | 0.0 |
| day238 | 40.0 | 5.91 | 0.0 | 0.0 |
| day 245 | 13.0 | 1.54 | 0.0 | 0.0 |
| day 267 | 23.0 | 1.13 | 0.0 | 0.0 |day 0: CAR-T therapy day
