# Supplementary material for: Rectum necrosis in a patient with severe COVID19 infection after CAR-T therapy: a case report
Source: Surg Case Rep. 2024 Sep 26;10:227. doi: 10.1186/s40792-024-02026-1 (PMC11427651; doi:10.1186/s40792-024-02026-1)
Supplement: Supplementary file 2 — Supplementary Material 2. [file 40792_2024_2026_MOESM2_ESM.pptx]

## Slide 1
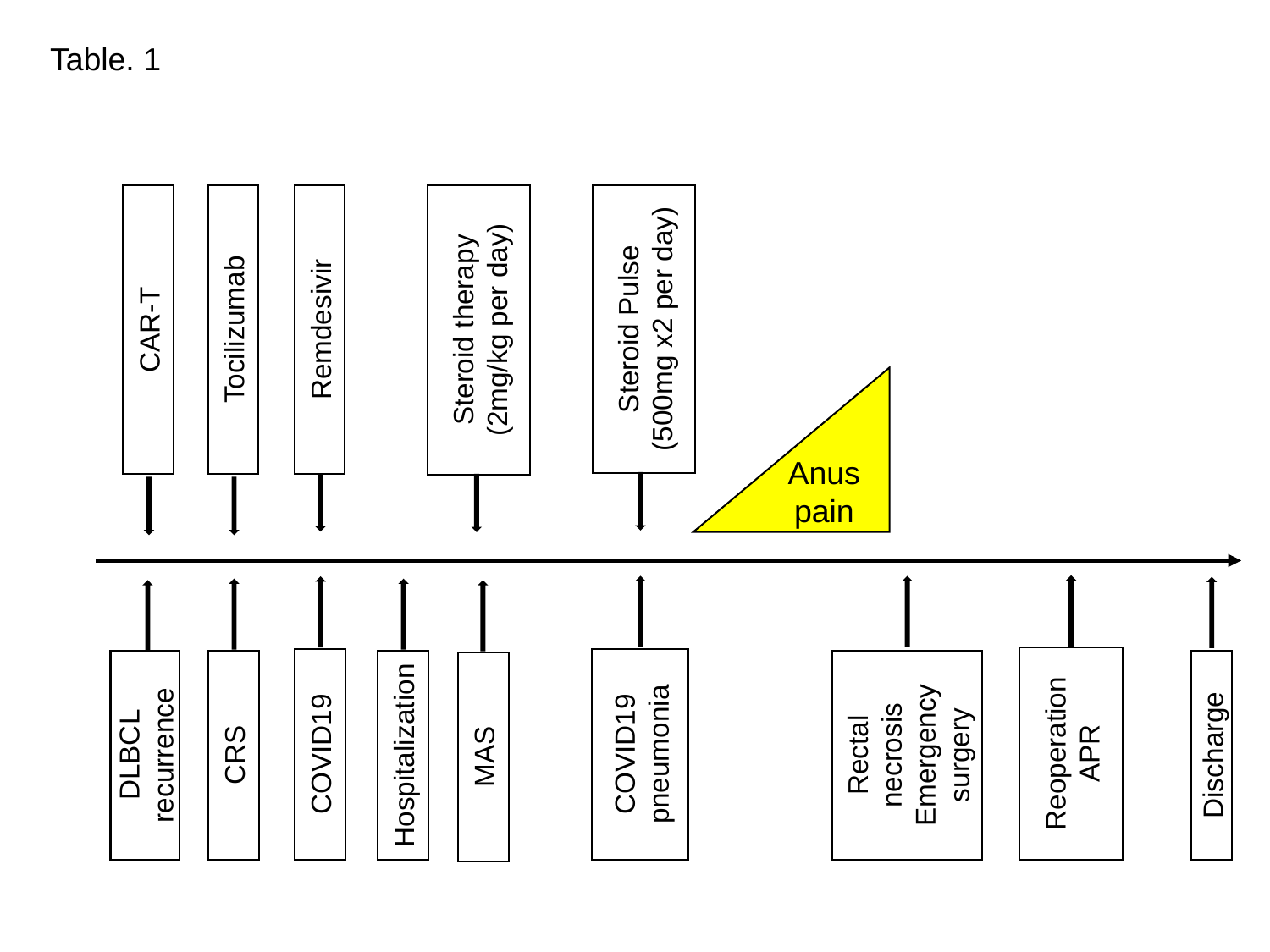

Table. 1
Steroid Pulse
(500mg x2 per day)
Steroid therapy
(2mg/kg per day)
CAR-T
Tocilizumab
Remdesivir
Anus pain
Rectal necrosis
Emergency surgery
Reoperation
APR
COVID19 pneumonia
DLBCL recurrence
COVID19
CRS
Hospitalization
MAS
Discharge
